# General Public Perception of Social Media, Impact of COVID-19 Pandemic, and Related Misconceptions

**DOI:** 10.1017/dmp.2021.229

**Published:** 2021-07-15

**Authors:** Rabiya Ali, Shireen Jawed, Mukhtiar Baig, Ahmad Azam Malik, Fatima Syed, Rehana Rehman

**Affiliations:** 1 Department of Physiology, Karachi Institute of Medical Sciences (KIMS), Karachi, Pakistan; 2 Department of Physiology, Aziz Fatima Medical and Dental College; 3 Department of Clinical Biochemistry, Faculty of Medicine, Rabigh, King Abdulaziz University, Jeddah, KSA; 4 Department of Family and Community Medicine, Faculty of Medicine, Rabigh, King Abdulaziz University, Jeddah, KSA; 5 University Institute of Public Health, The University of Lahore, Pakistan; 6 Aga Khan University, Karachi, Pakistan; 7 Department of Biological and Biomedical Sciences, Aga Khan University, Karachi, Pakistan

**Keywords:** COVID-19 impact, pakistani population, social media, misconceptions

## Abstract

**Objectives:**

This research aimed at investigating the general public perception of social media (SM), impact of COVID-19 (SARS-CoV-2) pandemic, and related misconceptions among the Pakistani population.

**Methodology:**

Cross-sectional study conducted during the peak of COVID-19 in Pakistan between May and June, 2020 comprised of 2307 Pakistani male and female participants. Subjects under 18 years of age and nationality other than Pakistani were excluded. An online questionnaire was administered via the Internet using various kinds of social media.

**Results:**

The study was comprised of 2307 male and female participants; 2074 (89.90%) used SM for seeking COVID-19 information, 450 (20%) used both Facebook (FB) and WhatsApp (WA), and 267 (11.6%) used FB, WA, Twitter, and Instagram. Respondents’ perceptions showed that: 529 (23%) believed in SM information and 1564 (67.8%) stated that COVID-19 affected their social and mental wellbeing. Respondents’ knowledge revealed that: 1509 (65.40%) had poor knowledge (≤ 50% score), and 798 (34.6%) had good knowledge (> 50% score) (*P* < 0.001) about COVID-19. Binary logistic regression analysis showed that higher-earning positively correlated, while private jobs were negatively associated, with good knowledge.

**Conclusion:**

FB and WA were the 2 common social media used by study participants (a third had good knowledge). COVID-19 affected the social, mental, and psychological well-being of individuals. Good knowledge was greater in individuals with higher earning and less with private job involvements.

## Introduction

Severe acute respiratory syndrome-coronavirus 2 (SARS-CoV-2) is an evolving situation that emerged from Wuhan, China.^1^ It became a global pandemic in a short duration of 4 months involving Pakistan.^2,3^ According to the World Health Organization, this deadly and life-threatening virus has affected 212 countries, infecting 72851747 individuals, and caused 1643339 fatalities worldwide.^4^

The exact cause of death due to this virus is still a dilemma, and the world is facing extensive loss due to this virus. This pandemic has put extensive pressure on the healthcare systems, economy, and governing bodies all over the world. Effective approaches by health professionals and governing bodies are required for the prevention of this lethal crisis.^5^ Lack of knowledge and misconceptions during these outbreaks increase the risk of COVID and further raises the burden on the health system.^6^

Rapid digitization is transforming almost everything from physical modes to software-controlled in every field including, banking, healthcare, and education systems. The digital landscape has grown enormously in Pakistan with a population of over 200000000 people. In Pakistan, mobile and internet connectivity is advancing the digital transformation of industries and educational institutes, and facilitating the development of new solutions especially in this corona scenario.^7^

In Pakistan approximately 165000000 people are mobile subscribers, 76380000 active internet users, and 60000000 Smartphone users. In this pandemic real time situation, the use of social media also increased especially during the lockdown; in Pakistan 37000000 people including children and elderly people, are currently using social media.^8^ Digitalization is paramount to establishing a modern, technology-led economy, and realizing the benefits of the Fourth Industrial Revolution.^7^ During lockdown, working at home became possible due to digitization which helped in reducing economical loss.^7^

Most people get information from SM due to its pivotal role in shaping the public’s knowledge, perceptions, and attitudes during various crises and outbreaks like COVID-19. WhatsApp, Facebook, Twitter, YouTube, and Instagram are the most popular and common SM sources that are used globally. Although SM is a powerful tool and source of fast dissemination of information addressing critical problems and health risks, this information is not always accurate.^6^ Information spread by various sites are not always credible, and it is difficult to distinguish rumors from reality. It has been reported by previous studies that public perception regarding social media is not trustworthy, yet despite that, posts rapidly circulate without people validating its authenticity, causing the rapid spread of misconception.^6,9,10^ Sometimes life-threatening misinformation is conveyed faster than the disease itself.^6^ Dangerous misinformation has been conveyed about the corona outbreak via social networks. This misleading information concerning different facets of the pandemic can threaten public safety and further aggravate this crisis. There is a need to correct misconceptions about health through SM. The public must refer to trustworthy sources for information concerning crises and diseases (e.g., WHO has provided social media teams for guiding the pandemic).^9^ Public awareness should be monitored regarding SM’s judicious use, which can be a useful tool for behavior and lifestyle modification during the COVID crises to promote social wellbeing and overcome this health challenge. The current study investigated the general public perception of social media, the impact of the COVID-19 pandemic, and related misconceptions. The novel coronavirus’ cases continue to increase in Pakistan and have surpassed an alarming figure of 600000.^11^ In a bid to manage this disastrous situation, the Government of Pakistan imposed a lockdown all over Pakistan that reduced the workforce across all economic sectors and caused unemployment. Limited workforce also reduced the productivity of the manufacturing sector leading to severe negative impact on Pakistan’s business and economy. A study  in Pakistan recommended  targeted mass psychological support programs to improve the mental health and well being of the population during the COVID-19 crises.^12^ According to a recent report of the United Nations Conference on Trade and Development (UNCTAD), Pakistan would be hardest hit by the global health crisis. Therefore, this justifies the need to carry out research concerning COVID-19 outbreak in Pakistan.^13^

## Methodology

This cross-sectional study was conducted during the peak of the COVID-19 pandemic in Pakistan between May and June 2020, at the Aziz Fatimah Medical and Dental College (AFMDC), Faisalabad, Pakistan. The AFMDC Institutional Ethical Committee (Reference No. 1EC /30-20) gave its ethical approval. Due to institutions’ closure and a dearth of all social activities, an online questionnaire was prepared and administered via the Internet using various SM. The questionnaire was developed using already published studies and the WHO myth-buster document.^14–16^ The questionnaire included demographic data and covered 3 themes: (1) General public perception of SM (3 questions), (2) The impact of COVID-19 pandemic on themselves, and their countries’ economic conditions (6 questions), and (3) COVID-19 related misconceptions (18 questions). Questions related to misconceptions had 3 options: yes/no/not sure. For each question, a score of 1 was given for the correct response and 0 for false and not sure. An individual score of 1-9 was taken as a low score (poor knowledge) while a score in the range of 10-18 was taken as a high score (good knowledge).^15^ The questionnaire was checked for its construct and content validity by 2 senior faculty members and was modified according to their suggestions.

A pilot study was then conducted on 40 people to determine their interpretation of the questionnaire and clear up any uncertainty. There were a few slight changes made accordingly. The Cronbach’s alpha tested the questions’ internal consistency and it was found to be 0.80.

Pakistan’s population is 212215000.^17^ The sample size was calculated using the Raosoft sample size calculator to obtain a representative sample from our target population by considering a 5% margin of error, 99% confidence level, and response distribution of 50%. The calculated sample size was 664. Nevertheless, to improve the study’s validity and generalizability, more people were included in the study. Male and female participants of age 18 years and above with Pakistani nationality were included in the study. Subjects under 18 years of age and nationality other than Pakistani were excluded. The sample’s demographic composition was similar to the general population of interest because our study had subjects from both genders, all socioeconomic conditions, all age groups, different educational, and job statuses. The questionnaire was rotated through emails, Facebook, Instagram, WhatsApp, etc. At the beginning of the questionnaire, a brief statement on consent and confidentiality was given, and we ensured the confidentiality of the data and participants’ identities. Filling the questionnaire implied permission to be included in the study; so no formal written permission was sought from participants.

The data was analyzed on SPSS-26. First, we determined the percentages and frequencies. We applied the score to misconception and transformed them into dichotomous variables by labeling good knowledge and poor knowledge according to participants’ scores. Percentages and frequencies were measured for impact and social media queries. *P* ≤ 0.05 was considered to be significant.

### Patient and Public Involvement

Patients were not involved in the current study. It was an online and population-based study. Data was collected from general Pakistani population. We will disseminate the study findings through email and WhatsApp.

## Results

**Figure 1.**
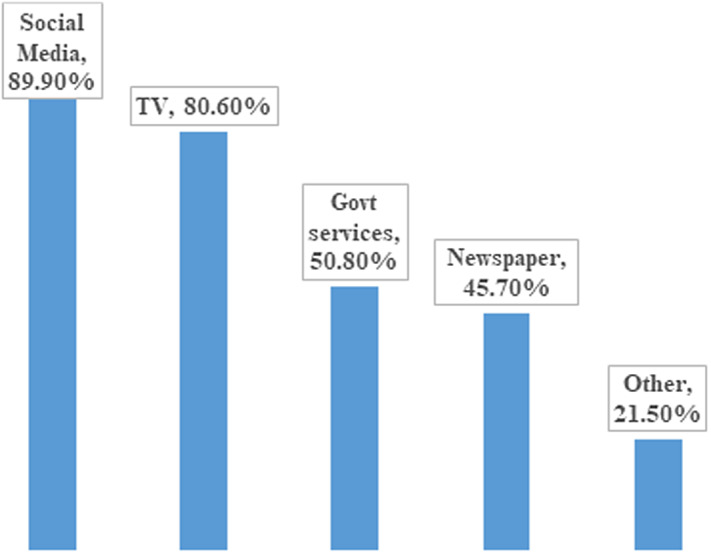
Percentages of participants using different information sources.

**Figure 2.**
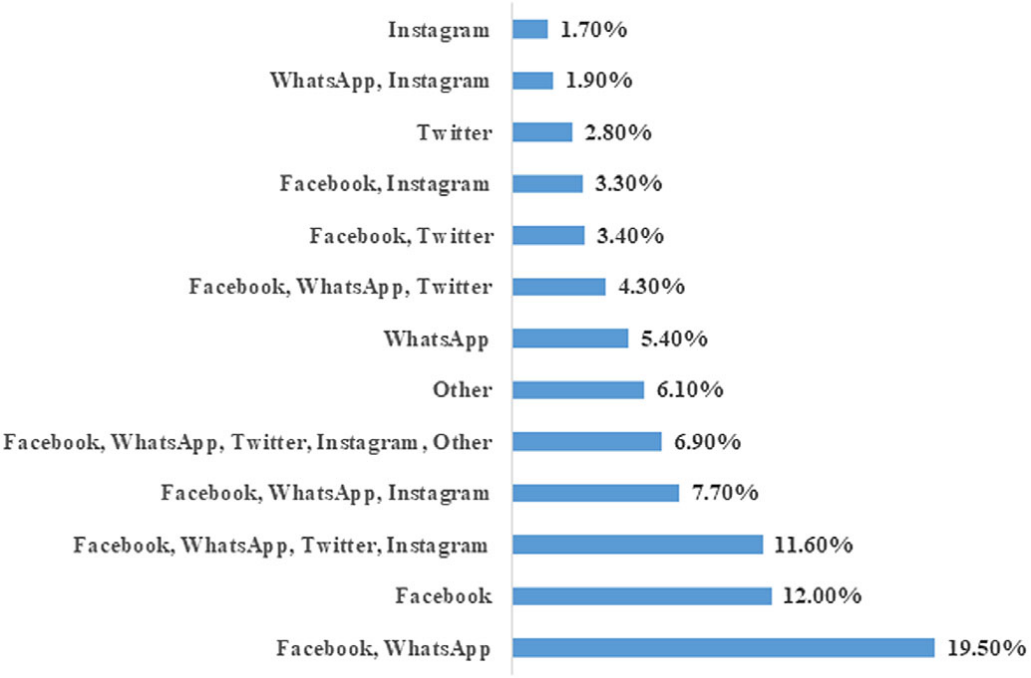
Percentage of participants using different social media.

**Figure 3.**
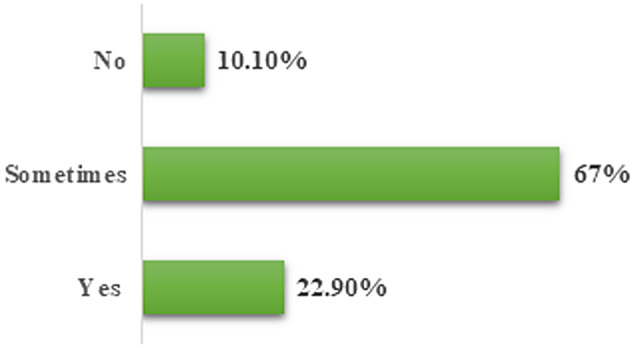
Do you believe in social media information?

This study was comprised of 2307 participants, of which 961 (41.7%) were females and 1346 (58.3%) were males. The participants were aged between 18 and 60 years. Most of the respondents used social media SM: 2074 (89.90%) for seeking COVID-19 information, followed by TV (1859, 80.6%), government services (1171, 50.8%), newspapers (1055, 45.7%), and others (495, 21.5%) (Figure 1). Out of the participants, 450 (20%) were using FB and WA, 267 (11.6%) were using FB, WA, TW, and Instagram (IG). About 178 (8%) were using FB, WA, and IG, while 160 (7%) were using FB, WA, TW, IG, and others. Several participants used a single type of social media: 277 (12%) used FB, 124 (5.4%) used WA, 64 (2.8%) used TW, and 40 (1.7%) used IG (Figure 2). Regarding the question, “Do you believe in social media information?” 529 (23%) responded yes, 1545 (67%) responded sometimes, and 233 (10%) said no (Figure 3).

Concerning the pandemic’s impact, almost 50% of the participants were ‘scared of Coronavirus’ and ‘scared of food shortage during the lockdown.’ 66.67% of the people stated that ‘COVID-19 is affecting their social, mental and psychological wellbeing’ (Table 1). The majority of the participants, 1813 (78.6%), believed that ‘this pandemic’s overall impact made them realize the importance of life.’ ‘I have become more religious during this pandemic’ was stated by 996 participants (43.2%). The majority of the participants thought that ‘the overall impact of this pandemic would be on the healthcare system, the economic condition of the people, and the country’ (Table 1).

**Table 1.** Study participants’ misconceptions and impact of COVID-19 pandemic

Statement (n = 2307)	Yes N (%)	No N (%)	Not sure N (%)
Vitamin C protects from COVID-19	1021(44.3)	620 (26.9)	665 (28.8)
An effective treatment is available against COVID-19	189 (8.2)	1508 (65.4)	610 (26.4)
This virus outbreak is only for a short period	232 (10)	1106 (47.9)	969 (42)
COVID-19 be spread through frozen food	235 (10.2)	971 (42.1)	1101 (47.7)
Eating garlic can prevent corona virus infection	296 (12.8)	1012 (43.9)	999 (43.3)
Females are more vulnerable to this infection	140 (6.1)	1294 (56.1)	873 (37.8)
Everyone should be tested for COVID-19	1106 (47.9)	875 (37.9)	326 (14.1)
Only older adults and younger people are at risk	448 (19.4)	1557 (67.5)	302 (13.1)
Cats and dogs spread Coronavirus	289 (12.5)	1285 (55.7)	733 (31.8)
Disposable face masks protect against Coronavirus	1423 (61.7)	381 (16.5)	503 (21.8)
Hand dryers kill Coronavirus	184 (8)	1304 (56.5)	819 (35.5)
You have to be with someone for 10 minutes to catch the virus	481 (20.8)	1113 (48.2)	713 (30.9)
Rinsing the nose with saline protects against Coronavirus	530 (23)	689 (29.9)	1088 (47.2)
Thermal scanners can diagnose Coronavirus	432 (18.7)	1119 (48.5)	756 (32.8)
Sipping water every 15 minutes can protect against corona infection	671 (29.1)	919 (39.8)	717 (31.1)
The Coronavirus will die off when temperature rises in the spring	226 (9.8)	1356 (58.8)	725 (31.4)
Coronavirus is the deadliest virus known to man	683 (29.6)	1141 (49.5)	483 (29.6)
Flu and pneumonia vaccines protect against COVID-19	127 (5.5)	1493 (64.7)	687 (29.8)

Impact questions	Yes	No	Not sure
I am very much scared of COVID-19 infection.	1116 (48.4)	902 (39.1)	289 (12.5)
I am scared of food shortage during lockdown	1122 (48.6)	986 (42.7)	199 (8.6)
COVID-19 is affecting my social, mental and psychological well being	1564 (67.8)	555 (24.1)	188 (8.1)

The overall impact of this pandemic on me: (participants were allowed to tick 1 or more options)			
I have realized the importance of life	1813 (78.6)		
I am more religious	996 (43.2)		
I have become careless	540 (23.4)		

The overall impact of this pandemic will on: (participants were allowed to tick 1 or more options)			
Healthcare system	1858 (80.5)		
Economic condition of the people	1964 (85.1)		
Economic condition of the country	2075 (89.9)		
Not sure	78 (3.4)		

Our results showed that there were several misconceptions among our study participants. A large number of our study participants (1021, 44.3%), believed that ‘intake of vitamin C protects from COVID-19’ and ‘Everyone should be tested for COVID-19.’About a third of the participants (671, 29.1%) believed that ‘sipping water every 15 minutes can protect corona infection,’ and ‘coronavirus is the deadliest virus known to man’ (Table 1). About 1 out of 8 (296, 12.8%) participants have a misconception that eating garlic can prevent coronavirus infection. A total of 140 (6.1%) subjects opined that females are more vulnerable to develop this infection, while 448 (19.4%) people thought that only older adults and younger people are at risk. About 289 participants (12.5%) believed that cats and dogs also spread this deadly infection (Table 1).

**Figure 4.**
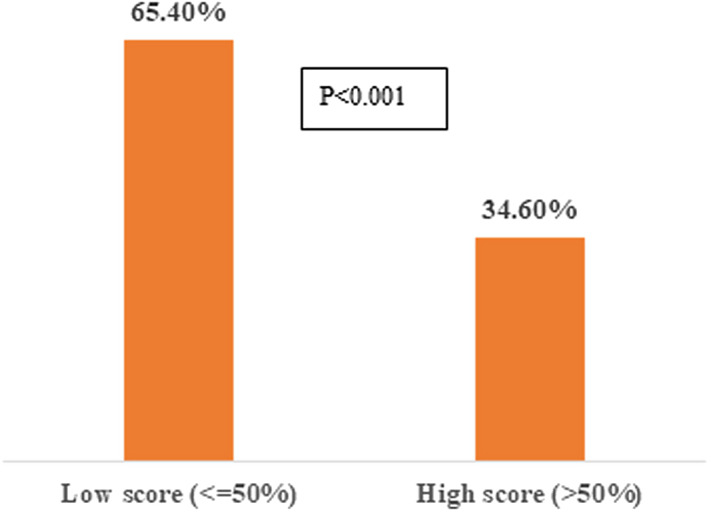
Comparison of misconceptions in study participants (low score indicated poor concepts and high scores indicated good concepts).

In our study, 66.67% of the participants (1509, 65.40%) had poor knowledge (≤ 50% score) compared to the 798 (34.6%) that had good knowledge (> 50% score) (*P* < 0.001) (Figure 4). Binary logistic regression analysis showed that people with higher earning (> 100000 PKR) was associated with a good concept (B = −0.238, OR = 2.642, *P* < 0.001) while the private job was negatively associated with good knowledge (B = −0.444, OR = 0.642, *P* = 0.001). Age groups, education, and marital status were not significantly associated with good knowledge (Table 2). Similarly, gender (OR = 1.096, *P* = 0.337) and information sources including SM and those who believed in SM were also not associated with good scores (OR = 1.283, *P* = 0.090).

**Table 2.** Predictors of good knowledge (binary logistic regression analysis)

Variables	B	S.E.	Wald	df	*P* - value	Odd Ratio	95% CI for EXP(B)	
							Lower	Upper
Gender								
Male vs Female	0.092	0.096	0.921	1	0.337	1.096	0.909	1.322
Age								
18 - 28 years	-0.373	0.350	1.139	1	0.286	0.689	0.347	1.366
29 - 39 years	-0.425	0.315	1.821	1	0.177	0.654	0.352	1.212
40 - 50 years	-0.526	0.331	2.533	1	0.111	0.591	0.309	1.130
51 - 60 years	-0.011	0.328	0.001	1	0.972	0.989	0.520	1.880
Income								
30000 - 50000 PKR	0.303	0.250	1.463	1	0.226	1.354	0.829	2.211
50001 - 100000 PKR	0.317	0.239	1.749	1	0.186	1.373	0.858	2.194
> 100000 PKR	0.971	0.238	16.655	1	0.000	2.642	1.657	4.212
None	0.271	0.194	1.952	1	0.162	1.311	0.897	1.916
Educational status								
High school degree	0.262	0.589	0.197	1	0.657	1.299	0.409	4.126
Bachelor’s degree	0.176	0.380	0.215	1	0.643	1.192	0.567	2.509
Master’s degree	-0.097	0.370	0.069	1	0.793	0.908	0.439	1.875
Professional degree	-0.279	0.379	0.541	1	0.462	0.757	0.360	1.591
PhD	-0.021	0.369	0.003	1	0.955	0.979	0.475	2.018
Job status								
Private job	-0.444	0.215	4.276	1	0.039	0.642	0.421	0.977
Business	-0.656	0.359	3.339	1	0.068	0.519	0.257	1.049
Housewife	-0.408	0.302	1.827	1	0.176	0.665	0.368	1.202
Student	-0.343	0.259	1.755	1	0.185	0.710	0.427	1.179
Marital status								
Unmarried	0.318	0.214	2.209	1	0.137	1.375	0.903	2.093
Divorced	1.053	0.591	3.171	1	0.075	2.866	0.899	9.133
Believe in SM								
Yes vs No	0.249	0.147	2.872	1	0.090	1.283	0.962	1.712
Information source								
TV	0.099	0.125	0.620	1	0.431	1.104	0.863	1.411
Newspaper	0.174	0.103	2.848	1	0.091	1.190	0.972	1.456
Social media	0.002	0.143	0.000	1	0.988	1.002	0.757	1.327
Govt. services	0.085	0.099	0.733	1	0.392	1.088	0.897	1.321
Others	0.019	0.112	0.029	1	0.864	1.019	0.819	1.269

## Discussion

The majority of the study respondents used social media (SM) (2074, 89.90%) for seeking COVID-19 information, followed by TV, government services, newspapers, and others. FB was the most common single social media used by most participants (12%), followed by FB, TW, and IG. The majority of the participants were using more than 1 type of social media, but surprisingly, only 23% believed the information from SM.

A close association has been observed between social media acquaintance and misperceptions about COVID-19. COVID-19 ﬁrst arrived in December 2019, and then the media became crazy with several misleading news about its contagious nature, mode of spread, infectious period, diagnostic approaches, and challenges.^18^ Due to this, the risk of info-demic was high as invalid or false information widely circulated on SM such as twitter, and they were considered as more popular than the valid information on news media.^18–21^

### Social Media and Misconceptions Concerning COVID-19

Our results showed that there were several misconceptions among our study participants. A large number of study participants, 1021 (44.3%), believed that ‘intake of vitamin C protects from COVID-19.’ This result is consistent with previous studies that mentioned that in the COVID-19 pandemic, due to strong attachment with SM, people developed a pseudoscientific belief that Vitamin C (Ascorbic acid) supplements are enough to prevent and treat COVID-19. Vitamin C is a powerful antioxidant and immunity booster that has long been lauded to protect against the influenza virus. Still, the clinical trials conducted thus far are unsuccessful in yielding unbiased results about the subject.^22^

The results of the current study revealed another misconception that ‘everyone should be tested for COVID-19.’ This result is consistent with the study that people assume that everyone should be tested for COVID-19.^23^ Nowadays, public attitude is dramatically affected by their perceptions of social media-related misconception.^23^ The world has encountered several queries regarding novel virus testing and diagnostic challenges: when, what, whom, or how to test? Several aspects are required to give satisfactory answers to these queries. It is a very unrealistic picture that everyone should be tested.^23^ During this serious crisis there is an urgent need for quick serological testing of frontline healthcare workers to show who has/had infection and is immune and therefore able to safely return to work.^24,25^ This will help in early recognition and management of true COVID patients to avoid crisis and hence reduce pressure on intensive care.^25,26^

Most of the participants of the current study believed that ‘Disposable face masks protect against Coronavirus.’ This result is consistent with the study that showed that a significant percentage of participants believed that common surgical masks are highly protective against infection from COVID-19.^27^ Dissemination of correct and relevant information drives may therefore, need to highlight the relative strength of: common surgical masks versus other means of prevention or control, particularly methodical handwashing or sanitization for an appropriate duration, avoiding contact with infected people, and by following the rules of social distancing.^27^ A recent Pakistani study also reported several misconceptions prevalent among the general population.^28^

In the current study, a third of the participants believed that ‘sipping water every 15 minutes can protect corona infection.’ This result is consistent with a study that mentioned that drinking warm water for every 15 to 20 minutes, or drinking lukewarm water with lemon impedes/prevents COVID-19, another false or pseudoscientific belief developed from social media.^29^ Regularly drinking warm water may develop a soothing effect on the sore throat,^30^ but there is no proof that drinking warm water alone or with lemon will prevent the infection.^31^ Misapprehension that drinking warm water will propel the COVID-19 virus from the pharynx to the esophageal tube then to the gastric lumen, where the gastric acid (HCl) destroys the virus.^30^ The COVID-19 virus is an enveloped virus that may cause an asymptomatic gastrointestinal infection. Usually, enveloped viruses cannot endure the gut’s rough milieu and promptly disintegrate with soaps and high heat and dryness. It is however, exciting to note that COVID-19 is unique in that it can bear the gut’s conditions.^30^ Concerning the pandemic’s impact and social media related misconception, almost 50% of the current study’s participants were ‘scared of coronavirus,’ and believed that ‘coronavirus is the deadliest virus known to man.’ This result is consistent with previous studies that mentioned that people are largely misinformed due to SM exposure, and dramatically overrate the contagiousness and mortality of COVID-19 comparable to specialists’ opinion.^27,28^ For example, on average, people assume that individuals may infect 28 others, while specialists believe that the range is between 1 – 6; it is also accepted that people who believe that COVID-19 is more infectious or fatal are least interested in obeying social distancing protocols (an observation regarded as Pandemic fatalism).^18,28,29^

### Impact of lockdown during COVID-19

According to the current study’s results, almost 50% of the participants were ‘scared of food shortage during the COVID-19 lockdown.’ This result is consistent with previous studies that accepted that food surety mainly gets undermined when demands reach a threshold that breaks the social and cultural bonds, thereby restricting families from sharing their rights.^32^ The impact is that stores of basic edible items in the market have progressively declined, leading to food shortages in lockdown phases. Poor people with low income, unemployed, and shelter-less people living on or below the margins, are chiefly exposed to food insecurity with extremely restricted access to sufficient food at economical rates.^33^

Most of the Pakistani population belong to the lower middle economic status and generally struggle to make ends meet. In this outbreak, the whole country was shut down, the people living below the poverty line are still at high risk of getting affected as they mostly rely on daily wages from informal work.^12^ In order to cope with this situation, the federal government has launched the Ehsaas program, which promised to provide financial assistance to the unemployed population.^34^

### Impact of COVID-19 on social well being

A total of 66.67% of the people in the current survey stated that ‘COVID-19 is affecting their social, mental and psychological well-being.’ This result is consistent with previous studies that mentioned the problems of mental trauma that people experienced, both due to restricted movement during the lockdown, and financial stress being faced by families, which has been persistently ignored and was largely unavailable in the current debate.^35^ The application of severe quarantine measures, isolation, academic institution closures, unemployment, financial burdens, and illness, all raise the risk for psychological distress. People have faced anxiety, episodes of panic disorders, and depression, most particularly with breaking news regarding the growing burden of COVID-19 patients at national and international levels.^35,36^

The majority of the participants (78.6%) in the current study believed that this pandemic’s overall impact made them realized the importance of life and religion; they become more religious” during this pandemic. This result is consistent with previous studies that documented that people believed that the disease could be cured through prayer and fasting.^19^ The scientists mentioned that despite high educational status levels, many erroneous beliefs and misconceptions were noted in the responses stemming from religious inclinations and the source of health information.^19^

### Impact of COVID-19 on economy and healthcare system

In the current study, most participants thought that ‘the overall impact of this pandemic would be on the healthcare system, the economic condition of the people, and the country.’ This result is consistent with previous studies that mentioned that strict lockdown imposed economic crises on families.^5^ As health and economy occupy the center stage in the current situation, a mutual struggle is needed on a large scale among public and private zones, which could be crucial for the public’s educational, health, economic, nutritional assistance and social restoration.^35^

Higher earning was positively associated, while private jobs were negatively associated with good knowledge. Interestingly, information sources including SM, and those who believed in SM were also not related to good concept scores. It reflects that people who use different information sources, including SM and those who believe in SM were more influenced by the misinformation disseminated via these sources. In the current study, SM was the main source of information related to COVID-19 infections, and 65% of the participants have poor knowledge concerning this crisis. Our binary logistic regression results justified this finding, which did not show any significant association of good knowledge with the information sources. In contrast to current results, a previous study conducted at Jordan University reported SM’s positive impact on good knowledge. Moreover, this Jordanian study also reported that SM has a crucial role in spreading correct information and improving the general population’s knowledge and attitudes concerning pandemics.^9^ Many websites are unauthentic, so in seeking valid information, authentic websites should be preferred.

### Impact of education level on knowledge concerning COVID-19

According to current research, no association has been observed between good knowledge and study participants holding a terminal PhD degree. A previous study reported that people with higher education tended to have more reasons to follow the valid guidelines, probably because of high self-awareness of their health.^36^ Contrary to these findings, another study documented that educated people have developed sufficient misconceptions about the current pandemic that the uneducated are likely to perceive as inaccurate information about COVID-19 and respond accordingly.^19^

Various previous studies conducted in Pakistan have been reported that a high proportion of their participants agreed that unauthentic information and fake news surfacing through social media regarding COVID-19 is having adverse impacts on their mental health and psychological wellbeing.^12^ Forwarding of unconfirmed information through posts or videos to the social media circle is a common practice in Pakistan.^37^

Social media is the informant which spreads myths about prevention and resistance against the disease.^30^ Spread of misinformation happens at a faster rate than that of actual fact through fast communication technologies which could be drastically damaging for the general public health, who follow this information without confirmation from any valid source. In order to avoid the misconceptions which are even more injurious than the disease itself, WHO has designed a web page ‘myth buster.’^31^ Perception of valid information is accepted to be critical in determining public awareness and accurate reaction to a global health emergency. Hence, it is crucially mandatory that the public have access to valid information that can inform them about the pandemic and what they should do to protect themselves, their families, and surroundings.^19^

Despite all types of myths and perceptions of its impact, it is postulated that immunization is the best way to combat the COVID-19 pandemic, and it is everyone’s social duty to get them vaccinated. It would not only ensure their protection, but would also assist in the prevention of its dissemination.^38^

## Limitations

Our study has a few limitations. We could not reach the people who were not using SM. Questionnaire-based studies are not free from respondents’ biases. Our research, because of its online nature, completely omitted illiterate people’s responses. In this real time corona situation, we were not able to administer questionnaires physically, so we shared our questionnaire among a large number of populations by using SM.

## Conclusion

FB and WA were the 2 common social media used by our study participants. Interestingly, less than a quarter of participants believed in SM information. Only a third of the study participants had good knowledge. Among 66.67% of the people stated that COVID-19 affected their social, mental, and psychological wellbeing. Higher earning was positively associated, while private jobs were negatively associated, with good knowledge. Information sources, including SM and those who believe in SM were also not associated with good concept scores. The information should be taken from an authentic and reliable source of information. For medically related information SM should be used carefully.

## Future Perspective

Misconceptions should be corrected in information drives conducted by authentic agencies. Hence health education is required to educate the population on a large scale and control the dissemination of misinformation circulated on social media.

The current investigation revealed proven facts for accurate public understanding and responses for COVID-19. In the absence of eﬀective remedy and vaccination against COVID-19, the pandemic can only be managed by circulating valid facts and news. The public should realize this and respond consequently. Better public understanding programs should be organized on hand sanitization, controlled mobility, and maintenance of social distancing, accepted as the only authentic proceedings that can control the current pandemic.

## Recommendations

People are advised to use social media wisely and intelligently to avoid any misconceptions. Authentic sites should be used for information about diseases. Social Media has an important role in the provision of authentic knowledge and improves the knowledge and awareness of the general public.
